# Spontaneous Regression of Metastatic Papillary Thyroid Cancer in a Lymph Node

**DOI:** 10.1155/2018/5873897

**Published:** 2018-03-20

**Authors:** Jien Shim, Jianyu Rao, Run Yu

**Affiliations:** ^1^Division of Endocrinology, Diabetes, and Metabolism, Department of Medicine, University of California, Los Angeles, David Geffen School of Medicine, Los Angeles, CA 90095, USA; ^2^Department of Pathology and Laboratory Medicine, University of California, Los Angeles, David Geffen School of Medicine, Los Angeles, CA 90095, USA

## Abstract

Spontaneous regression of cancer is defined as disappearance of cancer in the absence of specific therapy. In thyroid cancer patients with biochemically incomplete response to initial treatments, spontaneous decline in thyroglobulin levels without any cancer treatment is a well-known phenomenon; however, spontaneous regression of persistent or recurrent structural disease has not been reported. We here present a case of papillary thyroid cancer in a 58-year-old female who underwent total thyroidectomy and two radioiodine ablations. She had persistently elevated thyroglobulin levels. Six years after her initial treatments, she had biopsy-proven cervical lymph node metastasis. The patient opted not to undergo any further treatment. Over the course of the next 10 years, without any additional treatment, the lymph node disappeared and her thyroglobulin levels decreased to almost undetectable ranges, implying near-complete regression. Our case illustrates that metastatic papillary thyroid cancer in lymph nodes can regress spontaneously.

## 1. Introduction

Spontaneous regression of cancer is an extremely rare event where a malignant tumor disappears in the absence of anticancer treatment [1–3]. It has been reported in various types of cancer, but complete regression has not been reported in structurally evident papillary thyroid cancer. Here, we report a case of metastatic papillary thyroid cancer in a lymph node that regressed spontaneously.

## 2. Case Presentation

**Figure 1:**
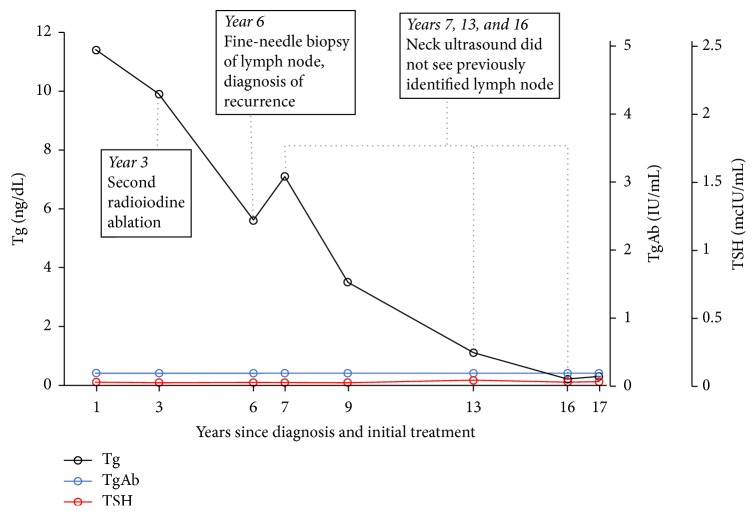
Time course of thyroglobulin (Tg), thyroglobulin antibody (TgAb), and TSH after initial treatment of papillary thyroid cancer.

A 58-year-old female with papillary thyroid cancer presented to the endocrinology clinic for follow-up. Patient had been first diagnosed with papillary thyroid cancer 6 years before at an outside clinic. She had undergone total thyroidectomy shortly after diagnosis, followed by radioiodine therapy with 150 mCi of I-131. She had postsurgical hypothyroidism, treated with levothyroxine 200 mcg orally daily, which suppressed her thyroid-stimulating hormone (TSH) to 0.02 mcIU/mL (reference range: 0.3–4.7). Levothyroxine dose was periodically titrated to keep TSH suppressed while keeping the patient clinically euthyroid. One year after total thyroidectomy and radioiodine therapy, she had persistently elevated thyroglobulin (Tg) level of 11.4 ng/dL (3–40) with undetectable thyroglobulin antibodies (TgAb) (Figure 1). She underwent a whole body scan with 4 mCi of I-131, which did not show any residual tumors in the thyroid bed or metastatic disease. Ultrasound of the neck did not detect suspicious lesions. Fluorodeoxyglucose positron emission tomography (FDG-PET) showed abnormal foci in the left lower anterior neck, right lower neck, and right upper neck, suspicious for metastatic lymph nodes. Magnetic resonance imaging (MRI) and computed tomography (CT) of the neck at the same time did not identify any abnormalities. The lymph nodes were characterized as benign appearing, the largest one measuring 16.7 mm on the left at level IB. Two years after radioactive iodine ablation, PET/CT again identified abnormal foci in the left lower neck and right upper neck, consistent with malignant lymph nodes. Three years after the first radioactive iodine ablation, patient's Tg was still elevated at 9.9 ng/dL with undetectable TgAb. Patient underwent second radioiodine ablation with 150 mCi of I-131. A whole body scan after I-131 radioiodine ablation did not show any abnormal activity.

**Figure 2:**
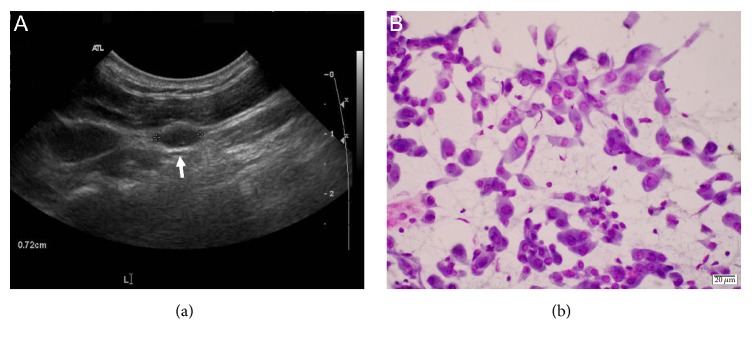
Metastatic papillary thyroid cancer in a lymph node. (a) Ultrasound image of the lymph node (arrow). (b) Cytological features of cells (Giemsa stain) obtained from the lymph node by fine-needle aspiration. Note the cytological features typical of papillary thyroid cancer cells. See text for detail.

At the endocrine clinic, 6 years after her total thyroidectomy and first radioactive iodine ablation and 3 years after the second radioactive iodine ablation, the patient felt well without dysphagia, dysphonia, or dyspnea. Her past medical history included type 2 diabetes. She had no family history of thyroid cancer. Her medications included aspirin 81 mg daily, sitagliptin-metformin 50–500 mg daily, and levothyroxine 137 mcg daily. Physical examination findings were unremarkable. Her serum Tg measured at 5.6 ng/mL with undetectable TgAb. TSH was <0.02 mcIU/mL and free T4 was 2.5 ng/dL (reference range 0.8–1.6 ng/dL). Neck ultrasound showed several lymph nodes on the left lateral neck and left submandibular region, measuring 0.5 cm to 0.8 cm (Figure 2). No abnormal masses were identified in the region of the thyroid bed. One of the enlarged lymph nodes measuring 0.8 cm in largest dimension was biopsied by fine-needle aspiration (FNA). Cytology showed moderately cellular, scattered clusters and sheets of moderately atypical epithelial cells with irregular nuclear contours, nuclear grooving, and numerous intranuclear pseudoinclusions, diagnostic of metastatic papillary thyroid carcinoma (Figure 2). Patient opted not to undergo surgery but rather to be monitored.

One year after the FNA, neck ultrasound no longer detects the previously identified abnormal appearing lymph nodes. Her Tg still measured 7.1 ng/mL with undetectable TgAb and suppressed TSH. The patient had sporadic follow-up with our clinic, but subsequent Tg levels over the next 10 years gradually declined (Figure 1) with undetectable TgAb and suppressed TSH. Neck ultrasound at 6 and 9 years after metastatic lymph node diagnosis (and 12 and 15 years after initial treatments with total thyroidectomy and first radioiodine ablation) did not identify any abnormalities, including lymphadenopathy.

## 3. Discussion

This case illustrates unprecedented spontaneous regression of papillary thyroid cancer metastasis in a lymph node. Spontaneous cancer regression is a rare phenomenon that has been reported in various types of cancers, including neuroblastoma, choriocarcinoma, malignant melanoma, lymphoma, renal cell carcinoma, and breast and colon cancer [1–3]. The mechanism of spontaneous regression is poorly understood, but a few of the proposed mechanisms include immune mediation, growth factor and cytokine-mediated mechanism, and hormonal mediation [4].

This is the first report of biopsy-proven metastatic differentiated thyroid cancer in a lymph node that regressed without any treatment. Differentiated thyroid cancer prognosis depends on the stage and the initial response to therapy during follow-up [5]. This is reflected in American Thyroid Association Dynamic Risk Stratification where individual cases are categorized as excellent response, biochemically incomplete response, structurally incomplete response, or indeterminate response [5]. Spontaneous remission of thyroid cancer in patients with biochemically incomplete response (but without structural disease) to initial therapy is an established phenomenon [6, 7]. Elevated Tg levels may spontaneously decline over time in patients without structural disease. Structural disease is defined as having imaging evidence of recurrence. In one study, after total thyroidectomy for differentiated thyroid cancer, 33.7% of patients with biochemically incomplete response had no evidence of disease after 10 years of follow-up, only managed with levothyroxine therapy with adequate suppression of TSH [6]. Spontaneous* partial* remission of metastatic papillary thyroid cancer has also been reported. High dose of I-131 therapy achieved long-term remission in 20 children exposed to Chernobyl disaster [7]. Children diagnosed with differentiated thyroid cancer with pulmonary metastasis underwent treatment with multiple I-131 ablation (total average dose 24.2 GBq, which is more than 600 mCi) in the initial 5 years after diagnosis; median Tg level was 5.6 ng/dL after the last I-131 treatment, but, 12 years later, more tban 10 patients had Tg levels <1 ng/dL without any additional treatment. With regard to lung metastases, all patients showed stable partial remission. This illustrates that metastatic disease of differentiated thyroid cancer may not progress and even regress over years without additional therapy. Spontaneous regression of structurally incomplete response to initial papillary thyroid cancer treatment is exceedingly rare. In the only study that we can find addressing the question, a single patient of the 153 (0.7%) with structurally incomplete response had no evidence of disease at final follow-up without any additional therapy, but no details of the patient were given [6]. Our patient had biopsy-proven papillary thyroid cancer grossly involving a lymph node that regressed within a year without any cancer treatment other than suppressive doses of levothyroxine. She had both biochemically and structurally incomplete response to initial total thyroidectomy and radioiodine ablation as shown by elevated nonstimulated Tg levels for many years and suspicious lymph node on ultrasound that was confirmed by cytology. To our knowledge, our patient represents the first described case in the literature on spontaneous, nearly complete regression of metastatic papillary thyroid cancer in lymph node or in any other organs. In particular, her structural disease had completely regressed on imaging at follow-up, unlike the children who had stable partial remission of lung metastases [7]. Surgical resection is typically the mainstay of treatment for recurrent structural thyroid cancer. In retrospect, aggressive management of our patient's isolated lymph node with surgery could have posed greater risks (e.g., perioperative risks and surgical complications) than observation alone. As seen in our case, active surveillance is a reasonable approach to small volume disease recurrence. Further studies are necessary to determine if observation alone has greater benefit in survival and quality of life than active treatment to eradicate the metastatic lesions, especially for stable, small volume recurrences.

Our case is one of a kind in the thyroid cancer literature, illustrating both a more rapid structural regression after lymph node biopsy and a slower biochemical regression of metastatic papillary cancer. Spontaneous regression of cancer after lymph node biopsy has been reported in other types of cancers. A metastatic non-small-cell lung cancer with primary tumor measuring 6 cm and mediastinal lymph node metastases regressed after paratracheal lymph node biopsy [8]. One theory to explain this phenomenon is that lymph node biopsy causes architectural destruction causing antigen release and triggering an immunological response [9]. The time course of cancer regression is usually within months of lymph node biopsy, similar to the course of spontaneous regression in our patient which happened within a year after fine-needle biopsy, making immunological mechanism in our case plausible. The spontaneous regression of papillary thyroid cancer in a lymph node in our case is different from partial primary tumor regression in papillary thyroid cancer [9]. First, the spontaneous regression in our case is virtually complete. Second, histologically, the lymph nodes in our case did not exhibit extensive fibrosis or lymphocytic infiltrate as shown in two cases of papillary thyroid microcarcinoma that regressed to less than 1.5 mm with metastasis to lymph nodes [10]. The slower biochemical regression of metastatic papillary cancer in our patient can be seen in 1/3 of patients with biochemical incomplete response to initial therapy, the mechanism for which remains unclear [6].

Spontaneous regression of metastatic papillary thyroid cancer in lymph nodes may not be very rare as currently believed. Patients with small recurrences may not have masses large enough to be detected on routine surveillance imaging, thus classifying them as biochemically incomplete rather than structurally incomplete. Perhaps spontaneous regression of differentiated thyroid cancer with structural metastases may be more common than we currently think. Our case further suggests that active surveillance is a reasonable approach to the management of small volume of recurrent metastatic papillary thyroid cancer.
